# Clinical Profile, Organ Dysfunction Patterns, and Outcomes in Paraquat Poisoning: A Retrospective Analysis

**DOI:** 10.7759/cureus.107106

**Published:** 2026-04-15

**Authors:** Mohammed Imaduddin Mehkri, Shristi Jayakumar Shetty, Bhargav V Bhat, Betsy Mathew, Nishanth Somashekar Ekambaranath

**Affiliations:** 1 General Medicine, Dr. Chandramma Dayananda Sagar Institute of Medical Education and Research (CDSIMER), Bangalore, IND

**Keywords:** acute kidney injury, mortality, multiorgan dysfunction, paraquat poisoning, respiratory failure

## Abstract

Background: Paraquat is a highly toxic herbicide associated with high mortality following acute ingestion, particularly in rural agricultural regions of India where it remains readily available.

Objective: To characterize the clinical profile and organ dysfunction patterns of acute paraquat poisoning and to describe their relationship with in-hospital mortality outcomes in a rural tertiary care setting.

Methods: This retrospective observational study included adults admitted with acute oral paraquat ingestion at a tertiary care hospital in South India between August 2020 and August 2025. Demographic data, clinical features, organ involvement, treatment modalities, and outcomes were analyzed.

Results: Twenty-seven patients were included (mean age, 33.4 ± 15.1 years; 20 males (74.1%)). All ingestions were intentional (27/27, 100%). Multiorgan involvement was common, with respiratory failure in 25 patients (92.6%), acute kidney injury in 21 (77.8%), circulatory failure in 20 (74.1%), and hepatic dysfunction in nine (33.3%). Overall in-hospital mortality was 20/27 (74.1%). Mortality increased progressively with the cumulative number of dysfunctional organ systems: no deaths occurred among patients with isolated single-organ dysfunction (0/4, 0%), mortality was three of six (50%) in those with two-organ involvement, and all patients with dysfunction of three or more organ systems did not survive in this cohort (17/17, 100%). Age, gender, and time to hospital presentation were not significantly associated with survival.

Conclusion: Acute paraquat poisoning is associated with severe multisystem toxicity and high mortality. Mortality increased markedly with increasing organ dysfunction burden, with uniformly fatal outcomes once three or more organ systems were involved. Early hemoperfusion, although unavailable at our center, has been associated with improved survival in prior studies. These findings underscore the need for improved institutional preparedness and consideration of strengthened regulatory measures in regions where paraquat remains widely accessible.

## Introduction

Paraquat (1,1'-dimethyl-4,4'-bipyridinium dichloride) represents one of the most acutely lethal pesticides in global agricultural use, with acute ingestion typically resulting in mortality rates exceeding 50%-90% [1]. The compound exerts its toxic effects through generation of reactive oxygen species, causing cellular damage via lipid peroxidation, mitochondrial dysfunction, and induction of apoptosis across multiple organ systems. In the notable absence of a specific antidote, therapeutic management remains fundamentally supportive in nature, targeting correction of dyselectrolytemia, management of multiorgan failure, and prevention of secondary complications [1].

The extreme toxicity profile of paraquat has prompted comprehensive regulatory action globally. More than 67 countries, including member states of the European Union, China, South Korea, and Taiwan, have implemented complete prohibitions [2]. Following Taiwan's comprehensive ban in 2007, paraquat-associated suicides declined by 58%, contributing to an overall 37% reduction in pesticide-related suicides [2]. Comparable public health benefits were documented in South Korea (46% reduction in pesticide suicides) and Sri Lanka (50% reduction) [2,3]. Importantly, implementation of paraquat prohibition has not resulted in any adverse effects on agricultural productivity in countries adopting such restrictions [4].

Despite these well-established global benefits, paraquat remains readily available within India and continues to serve as a preferred method for deliberate self-harm in rural agricultural communities [5]. Although some retrospective and prospective studies on paraquat poisoning have been done in large hospitals in India, there is still limited information about how patients in rural South India are affected especially in resource-limited centers with less access to advanced treatments like extracorporeal removal. This study aims to describe the clinical spectrum, patterns of organ dysfunction, and observed mortality trends in patients with paraquat poisoning admitted to a tertiary care center in South India, with a focus on clinically relevant bedside parameters rather than predictive modelling.

## Materials and methods

Study design and setting

A retrospective observational study was conducted at a tertiary care teaching hospital in Bangalore, Karnataka, serving a predominantly rural agricultural population. Ethical approval was obtained prior to data extraction. The study received approval from the Institutional Ethics Committee of Dr. Chandramma Dayananda Sagar Institute of Medical Education and Research (CDSIMER), Bangalore, India (Study Reference Number: CDSIMER/MR/0206/IEC/2025, dated: 24.03.2025).

Study population

Medical records of all patients admitted with acute paraquat poisoning between August 2020 and August 2025 were reviewed and identified by searching electronic health records for International Classification of Diseases, Tenth Revision (ICD-10) code T60.3 and cross-referencing these with Emergency and Critical Care Department admission logs. Inclusion criteria comprised adults aged ≥18 years with a documented history of acute oral paraquat ingestion. Patients with incomplete medical records or uncertain exposure history were excluded. Following the application of inclusion and exclusion criteria, a final cohort of 27 patients (n=27) was identified for inclusion in the study.

Data collection

Demographic data (age, gender, occupation), clinical features, laboratory investigations, radiological findings, management modalities, complications encountered, length of hospitalization, duration of stay in intensive care unit, and ultimate patient outcomes were systematically extracted using a standardized data extraction proforma. Data abstraction was performed by two independent reviewers to ensure accuracy, with any discrepancies resolved through consensus and review by a senior investigator. In cases of missing laboratory or clinical data, a 'complete-case analysis' approach was adopted, and the denominator for specific variables was adjusted accordingly.

Definitions

Acute kidney injury was defined according to Kidney Disease: Improving Global Outcomes (KDIGO) criteria as an increase in serum creatinine by ≥0.3 mg/dL within 48 hours, an increase to ≥1.5 times baseline within seven days, or urine output <0.5 mL/kg/h for at least six hours. Hepatic dysfunction was defined as transaminases exceeding three times the upper limit of normal or evidence of compromised synthetic function (prolonged prothrombin time, hypoalbuminemia). Respiratory failure was defined as hypoxemia requiring supplemental oxygen with clinical respiratory distress, non-invasive ventilation, or invasive mechanical ventilation. Circulatory failure was defined as systolic blood pressure less than 90 mmHg or requirement for vasopressor support.

Statistical analysis

Data were analyzed using IBM SPSS Statistics for Windows, version 27 (IBM Corp., Armonk, NY, USA). Categorical variables were presented as frequencies (%), and continuous variables as mean±standard deviation or median (interquartile range). Fisher's exact test was employed for categorical comparisons and the Mann-Whitney U test for continuous variables. A two-tailed p-value <0.05 was considered statistically significant. Due to the small sample size and complete separation of outcomes in the highest organ dysfunction category, multivariable regression modelling was not feasible. Alternative approaches such as penalized regression were considered; however, these were unlikely to yield stable estimates. Therefore, the analysis was primarily descriptive, with emphasis on clinically interpretable trends.

## Results

A total of 31 cases were identified, but four were excluded due to incomplete records and 27 met the inclusion criteria. Baseline demographic characteristics, organ dysfunction patterns, interventions, and outcomes were summarized in Table 1.

**Table 1:** Baseline demographic characteristics, organ dysfunction, interventions, and outcomes of patients with acute paraquat poisoning. Values are expressed as n (%) unless otherwise specified. Age is presented as mean ± standard deviation. ICU: intensive care unit.

Variable	n (%) / Mean ± SD
Age (years)	33.4 ± 15.1
Age 18-40	21 (77.8%)
Male sex	20 (74.1%)
Female sex	7 (25.9%)
Intentional ingestion	27 (100%)
Presentation <6 hours	18 (66.7%)
Presentation 6-24 hours	4 (14.8%)
Presentation >24 hours	5 (18.5%)
ICU admission	20 (74.1%)
Organ dysfunction	
Respiratory failure	25 (92.6%)
Acute kidney injury	21 (77.8%)
Circulatory failure (vasopressors)	20 (74.1%)
Hepatic dysfunction	9 (33.3%)
Number of organs involved	
1 organ	4 (14.8%)
2 organs	6 (22.2%)
≥3 organs	17 (62.96%)
Interventions	
Mechanical ventilation	20 (74.1%)
Hemodialysis	8 (29.6%)
N-acetylcysteine	15 (55.6%)
Corticosteroids	15 (55.6%)
Outcome	
In-hospital mortality	20 (74.1%)

Demographic and ingestion characteristics

The study cohort comprised 27 patients with a mean age of 33.4±15.1 years (median: 29 years, range: 18-85 years). Age distribution was concentrated in the younger demographic, with 21 patients (77.78%) aged 18-40 years, five patients (18.52%) aged 41-60 years, and one patient (3.70%) aged >60 years. The cohort was predominantly male, consisting of 20 male patients (74.1%) and seven female patients (25.9%), resulting in a male-to-female ratio of 2.86:1. Regarding the etiology of toxicity, all 27 cases (100%) involved intentional self-harm through deliberate paraquat ingestion, reflecting the prevalence of pesticide use as a common suicide method in rural agricultural communities. Precise quantification of the paraquat dose ingested was not reliable, as verbal histories suggested variable quantities and objective measurements were unavailable in the majority of cases; this precluded a formal dose-outcome correlation analysis. Furthermore, serum and urine paraquat levels were not available at our center during the study period.

Time elapsed between ingestion and hospital presentation

Early presentation (<six hours) was achieved in 18 patients (66.7%), intermediate presentation (six to 24 hours) in four patients (14.8%), and late presentation (>24 hours) in five patients (18.5%). The median time to hospital presentation was four and a half hours (interquartile range: 2-8 hours).

Mean length of hospital stay

Overall mean hospital stay was 6.4±5.0 days (median: 6 days, range: 1-18 days). Notably, survivors demonstrated longer hospitalization (mean: 10.0 days, range: 6-18 days) compared to non-survivors (mean: 5 days, range: 1-17 days). Six patients (22.2%) died within 24 hours of hospital admission. Mean intensive care unit stay was 4.4±3.4 days (median: 3 days, range 1-12 days), with 20 patients (74.1%) requiring intensive care unit admission.

Clinical presentation and organ involvement

Gastrointestinal manifestations were most common at presentation. Dysphagia was documented in 19 patients (70.4%), nausea/vomiting in 17 patients (63.0%), and oral ulceration in 15 patients (55.6%). Respiratory symptoms developed in 25 patients (92.6%), with dyspnea at the time of initial presentation in 18 patients (66.7%) and tachypnoea (respiratory rate >20 breaths per minute) in 16 patients (59.3%).

Acute kidney injury occurred in 21 patients (77.8%). Analysis of temporal progression revealed that 10 patients (37.0%) presented with acute kidney injury on the day of hospital admission (day 1), whilst 11 patients (40.7%) developed acute kidney injury after admission. Among those developing acute kidney injury post-admission: seven patients (25.9%) developed it on day 2, three patients (11.1%) on day 3, and one patient (3.7%) on day 4.

Hepatic dysfunction was noted in nine patients (33.3%). Laboratory assessment revealed elevation of transaminases (aspartate aminotransferase and alanine aminotransferase) exceeding three times the upper normal limit in these patients. Circulatory failure requiring vasopressor support developed in 20 patients (74.1%). Notably, only four patients (14.8%) presented with hypotension (systolic blood pressure: <90 mmHg) at initial assessment, whilst the remaining 16 patients (59.3%) required vasopressor support during the course of their hospitalization.

Number of organs involved

Based on cumulative organ dysfunction occurring at any time during hospitalization, four patients (14.8%) had isolated single-organ dysfunction, six patients (22.2%) developed dysfunction of two-organ systems, and 17 patients (62.96%) developed dysfunction of three or more organ systems (Table 1).

Treatment modalities

Mechanical ventilation was required in 20 patients (74.1%) for respiratory failure. Hemodialysis was done in eight patients (29.6%) for acute renal failure management. N-acetylcysteine was administered to 15 patients (55.6%) as antioxidant therapy, aligning with current recommendations for paraquat poisoning management [6]. Corticosteroids were administered to 15 patients (55.6%), reflecting ongoing uncertainty regarding immunosuppressive treatment efficacy.

Mortality

Total In-Hospital Mortality

The overall in-hospital mortality rate was 74.1% (n=20/27).

Age and Mortality

Age-stratified mortality analysis revealed: 18-30 years had 73.3% mortality (11/15 deaths); 31-50 years group had 62.5% mortality (5/8 deaths); and >50 years had 100% mortality (4/4 deaths). However, the association between age and mortality was not statistically significant, Mann-Whitney U test, (p=0.638). No statistically significant association between age and mortality was observed.

Time to Presentation and Mortality

No association between time to presentation and survival was observed (p=0.939), with 13 of 18 (72.2%) mortality in early presenters versus four of five (80.0%) in late presenters.

Organ Failure and Mortality

Respiratory failure was observed in 25 patients (92.6%), including the majority of non-survivors. All patients requiring mechanical ventilation (20/20) did not survive during hospitalization, reflecting advanced disease severity in this subgroup. Acute kidney injury was observed in 21 patients (77.8%) and was more frequent among patients who did not survive (19/21). Circulatory failure requiring vasopressor support was observed in 20 patients (74.1%), all of whom did not survive, indicating advanced disease severity in this subgroup. Hepatic dysfunction was observed in nine patients (33.3%), with seven of these patients not surviving during hospitalization.

Mortality and Number of Dysfunctional Organs

**Figure 1:**
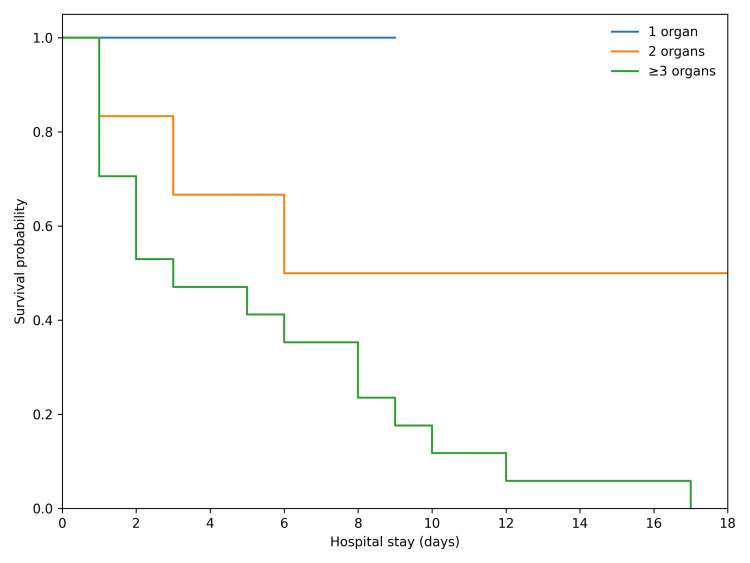
Kaplan-Meier survival curves stratified by cumulative number of dysfunctional organ systems in acute paraquat poisoning. Organ dysfunction was defined as acute kidney injury, respiratory failure, hepatic dysfunction, or circulatory failure occurring at any time during hospitalization. Survival probability declined progressively with increasing organ failure burden, with uniformly fatal outcomes observed in patients developing dysfunction of three or more organ systems in this cohort.

Mortality increased markedly with increasing organ dysfunction burden. No deaths occurred among patients with isolated single-organ dysfunction (0/4, 0%). Among patients with two-organ involvement, mortality was three of six (50%), while all patients 17 of 17 (100%) who developed dysfunction of three or more organ systems died (Table 2). Kaplan-Meier survival analysis further demonstrated progressively reduced survival probability with increasing cumulative organ dysfunction (Figure 1).

**Table 2:** Number of dysfunctional organs and mortality (n = 27). Values are expressed as n (%). Mortality indicates in-hospital mortality.

Number of dysfunctional organs	Patients (n)	Mortality n (%)
1	4	0 (0)
2	6	3 (50)
≥3	17	17 (100)

Treatment Modalities and Mortality Outcomes

All 20/20 patients (100%) who required mechanical ventilation in this cohort died. Hemodialysis was done in eight patients (29.6%) for acute renal failure. Mortality in this subgroup was 75% (6/8 patients). N-acetylcysteine was given to 15 patients and only three survived (80% mortality). Fifteen patients received corticosteroids and mortality in this subgroup was 11/15 (73.3%). No statistically significant difference in mortality was observed among the treatment modalities evaluated.

## Discussion

**Figure 2:**
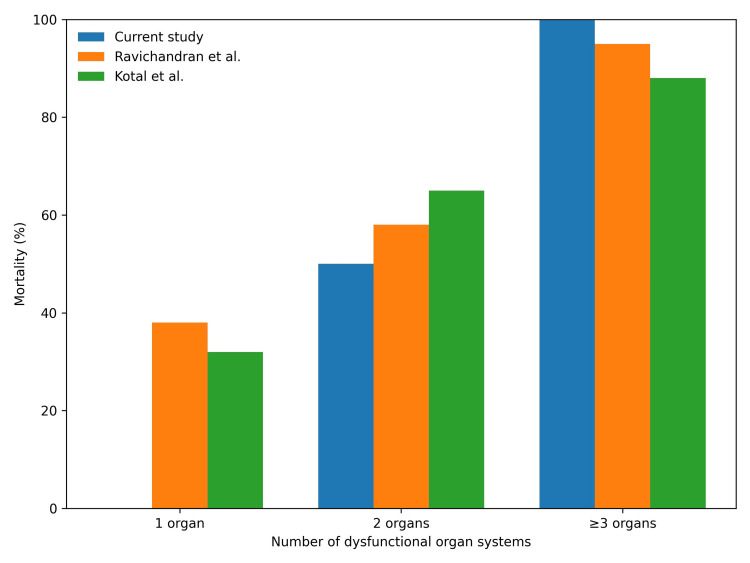
In-hospital mortality according to the number of dysfunctional organ systems in the present cohort compared with selected published cohorts. Bars represent in-hospital mortality percentages stratified by number of dysfunctional organ systems. Data from previously published cohorts (Ravichandran et al. [3] and Kotal et al. [10]) are shown for descriptive comparison only and were not subjected to statistical testing.

This retrospective study highlights the high lethality of acute paraquat poisoning, with an in-hospital mortality of 20/27 (74.1%), consistent with the upper range of mortality reported globally (33%-91.7%) [3,4,7]. The most clinically relevant finding is the graded increase in mortality with increasing organ dysfunction burden, culminating in 17/17 (100%) mortality when three or more organ systems were involved (Figure 2). This pattern is consistent with findings from multiple Indian and international cohorts, which similarly demonstrate progressive mortality with increasing organ dysfunction burden (Figure 2) [3,7-9].

The absence of survivors among patients with three or more organ dysfunctions in this cohort highlights the severity of advanced multiorgan failure. The demographic profile of predominantly young adult males is consistent with published literature from India and other agricultural regions, including reports from Southern and Northeast India, reflecting occupational access and intentional self-harm patterns [3,8,10]. Similar to earlier reports, age and gender were not statistically significant predictors of mortality in our cohort [3,7,11], suggesting that paraquat toxicity may outweigh baseline demographic or physiological differences.

Respiratory involvement was nearly universal in our study 25/27 (92.6%) and was observed in all patients who did not survive and required mechanical ventilation, although prior studies report mortality rates of 72%-82% among ventilated patients, the 20/20 (100%) mortality observed in our cohort likely reflects particularly severe lung injury, delayed toxin clearance, and absence of early hemoperfusion [3,4]. Paraquat’s selective pulmonary accumulation through the polyamine uptake system and subsequent progression from pneumonitis to fibrosis is well documented and explains the profound respiratory lethality observed [1,6].

Acute kidney injury occurred in 21/27 (77.8%) patients, comparable to rates reported in other Indian and international cohorts of acute paraquat poisoning [3,7-9]. The early onset of renal dysfunction in our cohort supports the established mechanism of direct proximal tubular toxicity. Consistent with prior studies and meta-analyses, acute kidney injury was associated with very high mortality and has been identified as an early prognostic marker in paraquat poisoning, reflecting both toxin burden and evolving multiorgan dysfunction [11,12].

Circulatory failure requiring vasopressor support was observed in 20/27 (74.1%) patients and was associated with 20/20 (100%) mortality, supporting shock as a marker of advanced disease and extremely poor prognosis in paraquat toxicity. This mortality rate exceeds that reported in earlier series, where circulatory failure was less frequent and less uniformly fatal [3,4]. The development of shock likely reflects widespread endothelial injury, myocardial depression, and severe metabolic derangements in advanced poisoning. The 100% mortality in this subgroup suggests a physiological 'point of no return,' where conventional vasopressor support is inadequate to bridge patients through the peak phase of multiorgan dysfunction. Hepatic dysfunction, observed in one-third of patients, occurred less frequently than pulmonary or renal involvement but was still associated with substantial mortality. Similar rates have been reported across studies, with hepatic injury contributing to impaired detoxification capacity and worsening oxidative stress [3,4,10]. 

Despite early hospital presentation in two-thirds of patients, no survival benefit was observed. This finding aligns with prior studies demonstrating that early presentation alone does not improve outcomes unless coupled with early hemoperfusion [13]. Hemoperfusion, when initiated within six hours of ingestion, has consistently shown survival benefits, with reported mortality reductions from over 90% to approximately 40%-60% [12-14]. The absence of hemoperfusion capability at our institution may have contributed to the high mortality observed. This suggests that the 'golden hour' for gastric decontamination is likely insufficient to prevent irreversible systemic toxicity unless followed by rapid extracorporeal removal to mitigate the compound's rapid distribution into the pulmonary parenchyma.

Adjunctive therapies such as N-acetylcysteine and corticosteroids were frequently used but did not demonstrate clear survival benefit, consistent with existing systematic reviews and meta-analyses showing inconsistent or modest effects [6,14,15]. Conventional hemodialysis, while essential for renal failure management, remains ineffective for paraquat clearance due to the compound’s rapid tissue distribution and large volume of distribution, explaining the limited impact on survival seen in our cohort [5].

Overall, our findings are concordant with published evidence indicating that once multiorgan failure develops, particularly involving the lungs and circulatory system, prognosis is extremely poor regardless of supportive interventions [3,4,7,16]. These consistently high mortality rates across diverse settings underscore the substantial clinical and public health burden of paraquat poisoning and support calls for stronger regulatory measures, as previously advocated in multiple countries, where such measures have been associated with substantial reductions in suicide mortality without adverse agricultural impact [2,5,17,18]. From a health system standpoint, improving institutional preparedness, including development of hemoperfusion capability, earlier recognition of multiorgan dysfunction, access to specialist toxicology expertise, and integration of community-based suicide prevention strategies, may help to mitigate the high mortality associated with paraquat poisoning in affected regions.

Limitations of study

Several limitations warrant acknowledgment. Primarily, the retrospective design and single-center nature of this study may constrain the generalizability of the findings to different geographic regions and healthcare settings. The modest sample size (n=27), while providing clinical insights, limits the statistical power to detect associations for less common outcomes; consequently, findings should be interpreted as descriptive rather than predictive.

The absence of hemoperfusion at our facility represents a significant treatment limitation that may affect the comparability of our outcomes with centers utilizing this modality. Furthermore, quantification of the paraquat dose was not reliably documented, and serum or urine paraquat concentration measurements were unavailable. This precluded the calculation of validated prognostic tools, such as the Severity Index of Paraquat Poisoning (SIPP), and prevented formal dose-outcome correlation analyses.

Additionally, standard severity indices, such as Sequential Organ Failure Assessment (SOFA) or Acute Physiology and Chronic Health Evaluation II (APACHE II) scores, could not be consistently calculated due to the retrospective nature of data collection. The institutional setting may also introduce referral bias, potentially overrepresenting severe cases. Furthermore, six patients died within 24 hours of admission, which may lead to survivorship bias and an incomplete capture of organ dysfunction trajectories. Furthermore, the lack of a standardized treatment protocol, with management decisions left to the discretion of the treating clinical team, introduces potential variability in the supportive care received by patients. Finally, the lack of systematic long-term follow-up for survivors limits our assessment of chronic morbidity, particularly regarding persistent pulmonary dysfunction.

## Conclusions

This retrospective analysis highlights the severe systemic toxicity and high in-hospital mortality associated with acute paraquat ingestion. Early presentation alone was not associated with improved survival in our cohort in the absence of early hemoperfusion capability. The findings add to accumulating evidence supporting consideration of strengthened regulatory measures in regions where paraquat remains readily accessible.

This position is reinforced by consistent reports of high mortality across Indian and international cohorts, alongside evidence from multiple countries that have implemented paraquat restrictions with subsequent reductions in pesticide-related suicide without adverse agricultural impact. Implementation of such regulatory measures may contribute to reducing the burden of paraquat poisoning and represent an important public health strategy in agricultural communities.
